# Molecular Mechanisms of Bortezomib Resistant Adenocarcinoma Cells

**DOI:** 10.1371/journal.pone.0027996

**Published:** 2011-12-22

**Authors:** Erika Suzuki, Susan Demo, Edgar Deu, Jonathan Keats, Shirin Arastu-Kapur, P. Leif Bergsagel, Mark K. Bennett, Christopher J. Kirk

**Affiliations:** 1 Onyx Pharmaceuticals, South San Francisco, California, United States of America; 2 Stanford University, Stanford, California, United States of America; 3 Translational Genomics Research Institute, Phoenix, Arizona, United States of America; 4 Mayo Clinic, Scottsdale, Arizona, United States of America; The Ohio State University, United States of America

## Abstract

Bortezomib (Velcade™) is a reversible proteasome inhibitor that is approved for the treatment of multiple myeloma (MM). Despite its demonstrated clinical success, some patients are deprived of treatment due to primary refractoriness or development of resistance during therapy. To investigate the role of the duration of proteasome inhibition in the anti-tumor response of bortezomib, we established clonal isolates of HT-29 adenocarcinoma cells adapted to continuous exposure of bortezomib. These cells were ∼30-fold resistant to bortezomib. Two novel and distinct mutations in the β5 subunit, Cys63Phe, located distal to the binding site in a helix critical for drug binding, and Arg24Cys, found in the propeptide region were found in all resistant clones. The latter mutation is a natural variant found to be elevated in frequency in patients with MM. Proteasome activity and levels of both the constitutive and immunoproteasome were increased in resistant cells, which correlated to an increase in subunit gene expression. These changes correlated with a more rapid recovery of proteasome activity following brief exposure to bortezomib. Increased recovery rate was not due to increased proteasome turnover as similar findings were seen in cells co-treated with cycloheximide. When we exposed resistant cells to the irreversible proteasome inhibitor carfilzomib we noted a slower rate of recovery of proteasome activity as compared to bortezomib in both parental and resistant cells. Importantly, carfilzomib maintained its cytotoxic potential in the bortezomib resistant cell lines. Therefore, resistance to bortezomib, can be overcome with irreversible inhibitors, suggesting prolonged proteasome inhibition induces a more potent anti-tumor response.

## Introduction

The proteasome is a multicatalytic proteolytic structure that is responsible for the degradation of intracellular proteins [1]. Three distinct catalytic activities comprise the proteasome: chymotrypsin-like (CT-L), caspase-like (C-L), and trypsin-like (T-L). These activities are encoded in the broadly expressed constitutive (c20S) form of the proteasome by β5, β1, and β2, respectively. Another form of the proteasome that is primarily expressed in cells of hematopoietic origin and cells exposed to inflammatory cytokines, known as the immunoproteasome (i20S), has the three catalytic activities represented by LMP7, LMP2, and MECL1. In cells that express both types of proteasomes, hybrid ensembles containing both c20S and i20S catalytic subunits have been described [2].

Proteasome inhibition leads to the accumulation of unfolded or oxidatively modified proteins in the intracellular environment, which causes an overload in the endoplasmic reticulum (ER). The ER stress response initially induces a pro-survival response by activating the unfolded protein response (UPR) to promote refolding or elimination of unfolded proteins [3]. Three signaling modules of the unfolded protein response are activated: *i*) Regulation of translation by PERK activation of ATF4, *ii*) regulation of proteolysis by cleavage ATF6 and translocation to the nucleus, and *iii*) transcriptional control by IRE1 splicing of XBP1u mRNA. If pro-survival mechanisms are overwhelmed by protein overload or are malfunctioning, apoptosis is induced [3]. In B-cell neoplasms, targeting the proteasome with small molecule inhibitors has led to new therapeutic strategies. The first proteasome inhibitor to gain FDA approval is the reversible dipeptide boronate boretezomib (Velcade™), which is approved for the treatment of multiple myeloma (MM) and mantle cell lymphoma [4]. The clinical success of bortezomib has led to the development of several other small molecule inhibitors encompassing multiple chemical classes [5], [6].

Despite the overwhelming success of bortezomib in the treatment of MM, a subset of bortezomib naïve patients fail to respond to therapy and others develop resistance upon relapse [7], [8]. The study of resistance to bortezomib has involved gene expression profiling in patient derived tumor cells [9] and the generation of cell lines with acquired resistance to bortezomib [10]–[15]. Increased expression of the ER stress response pathways has been noted in both settings [9], [16]. Whether these mechanisms of resistance are specific to bortezomib or a general route of defense against proteasome inhibitor pressure is unclear. In addition, increased expression levels of the proteasome [11], [13], [15], [17], [18] and mutations in the β5 subunit [12], [13], [17], [19], the primary target of bortezomib, have been described in cells adapted to bortezomib in vitro. However, no mutations in β5 have been detected in myeloma patients refractory to or relapsed from bortezomib treatment [20].

We describe here the generation of bortezomib resistant cells in HT-29 adenocarcinoma cells to investigate the role of proteasome duration in cytotoxicity. We chose a solid tumor cell line to pursue our studies since the dynamic window between complete proteasome inhibition and cytotoxicity is larger than in hematologic tumor cell lines [21]. The clonal isolates conditioned to bortezomib displayed a stable resistance of 30–60 fold relative to parental cells These cells were globally analyzed for expression and genetic changes and specifically assessed for biochemical, expression, and genetic changes to proteasome active sites. We found that these resistant cells had a novel mutation within the mature β5 and a mutation in the propeptide region of the β5 subunit which is a natural variant found at a higher frequency in multiple myeloma patients. The resistant cells also had an increased subunit expression of multiple proteasome subunits that resulted in cells that had higher basal proteasome activity. Further, these cells recovered proteasome activity more rapidly following brief exposure to bortezomib. Interestingly, we found that proteasome inhibitors with an irreversible mechanism of action could overcome bortezomib resistance in these cells, suggesting that prolonged inhibition of the proteasome induces a more potent cytotoxic response to tumor cells.

## Materials and Methods

### Cell culture

HT-29 cells (American Type Culture Collection, Manassas, VA) were cultured in 37°C incubators with 5% CO_2_, using McCoy's 5a with L-glutamine and supplemented with 10% fetal bovine serum (FBS), 2.2 g/l sodium bicarbonate, 100 units/ml penicillin, 100 units/ml streptomycin (Mediatech; Manassas, VA) and 1.5 g/l D-glucose (Sigma Aldrich; Carlsbad, CA). Drug treatment media used in assays was composed of McCoy's 5a with L-glutamine supplemented with 10% FBS, 100 units/ml penicillin and 100 units/ml streptomycin. Both parental and bortezomib-conditioned HT-29 cells were maintained as adherent cells in corning 175 mm^2^ flat bottom flasks. These adherent cells were passaged using trypsin/EDTA (Mediatech) and plated at 4.0×10^5^ cells/cm^2^.

### Reagents

Cycloheximide was purchased from Enzo Life Sciences (Ann Arbor, MI) and dissolved to a stock concentration of 25 mg/ml in DMSO and further diluted to 1 mg/ml for cell culture experiments. Carfilzomib was manufactured at Onyx Pharmaceuticals, Inc. Bortezomib (Velcade®; Millennium Pharmaceuticals) was purchased from a local pharmacy. Carfilzomib was dissolved in dimethyl sulfoxide (DMSO; Sigma Aldrich; Carlsbad, CA) to a stock concentration of 10 mM while bortezomib was dissolved in saline (0.9%; Sigma Aldrich) to a stock concentration of 2.6 mM. Both compounds were further diluted in DMSO to their respective working concentrations with a final concentration of DMSO at 0.25% in each experimental condition. Cbz-Leu-Leu-Leu-Boronic acid was obtained from AG Scientific, Inc. (San Diego, CA) and dissolved in DMSO to a final concentration of 10 mM. The reconstituted stock compounds were dispensed into single-use aliquots and stored at −80°C until use.

### Isolation of bortezomib-resistant single cell clones

HT-29 cells were cultured with continuous stepwise increases in bortezomib concentration (20 nM to 200 nM) over 7 months. Clonal isolates were derived by 2 separate limiting dilution analyses in 96-well plates under constant bortezomib exposure of 100 nM or 200 nM for an additional 4 months.

### Cell viability assay

Cell viability was measured after 72 hr of continuous drug treatment using CellTiter-Glo® (Promega Corp.; Madison, WI) according to the manufacturer's instructions. Clonal populations stably growing under bortezomib pressure were harvested, washed to remove free bortezomib to allow proteasome recovery, then plated in 96-well plates (3×10^4^ cells/well) for 3–40 days prior to the assay day; parental HT-29 cells (no prior exposure to bortezomib) were assayed in parallel. Serial dilutions of bortezomib or carfilzomib were added to replicate wells to obtain dose responses. Cell viability experiments were performed in triplicate for each cell line. Viability (average of duplicate determinations at each dose) was then plotted against drug concentration for each drug/cell line combination, and the best-fit curve was determined using a four-parameter (sigmoidal) model. Data analysis was performed using GraphPad Prism (v5; San Diego, CA).

### Western blot analysis

HT-29 parental and bortezomib-resistant cells were cultured as described above. At different time points, cells were harvested and cells were lysed in 0.2% Triton X-100 with protease inhibitor cocktail (Roche; South San Francisco, CA). Lysates were normalized by protein assay then resolved on NuPage gels (Invitrogen; Carlsbad, CA), transferred to nitrocellulose, and probed with antibodies to β1, β2, β5, LMP2, LMP7 and MECL1. HRP-conjugated secondary antibodies were used to detect the immunoreactive bands, followed by chemiluminescence detection (Thermo Scientific; Rockford, IL).

### 20S proteasome activity assay

Cells were incubated for 1 hr with serial dilutions of carfilzomib or bortezomib followed by washing with media and allowed to recover their proteasomes for 1, 2, 4, 6, 8, 12 or 24 hr or immediate washed with phosphate buffered saline (PBS) and resuspended in 30 µl lysis buffer (20 mM Tris pH 8 and 5 mM EDTA). Cells were kept frozen at −80°C until use. The day of the assay, cells were thawed on ice, centrifuged at 3000 rpm for 20 min and the supernatants were combined with the specific fluorogenic substrate in a 384-well plate. The chymotrypsin-like, trypsin-like, and caspase-like activities of the cellular 20S proteasomes were determined by measuring the appearance of a fluorescent cleavage product generated from the fluorogenic substrates Suc-LLVY-AMC, Bz-VGR-AMC, and Z-LLE-AMC (Boston Biochem Inc., Cambridge, MA), respectively. The samples were analyzed on a spectrofluorometer (Tecan Safire; San Jose, CA), using an excitation of 380 nm and an emission of 460 nm.

### Proteasome active site ELISA

Bortezomib resistant cells were cultured without bortezomib for at least 3 days, then harvested and lysed in lysis buffer as described above. A proteasome active site ELISA was used to determine the levels of constitutive and immunoproteasome active sites and was performed as described previously [16]. Briefly, protein normalized samples (lysed cells) were incubated with a biotinylated active site probe PR-584 (5–15 µM) for 2 hrs in a 25°C water bath. Streptavidin-sepharose beads (GE Healthcare, Sweden) (2.5–5 µL packed beads per well) were added to 96-well filter plates (Multiscreen DV; Millipore, Billerica, MA) followed by 70 µL of 8 M guanidine (Sigma Aldrich) per well to serve as a denaturant for the samples. Samples were added to the beads and guanidine for 1 hr at room temperature on a plate shaker. The beads were washed 5 times with 200 µl/well of ELISA buffer (PBS, 1% bovine serum albumin, 0.1% tween-20) by vacuum filtration. The beads were incubated overnight at 4°C on a plate shaker with the following antibodies recognizing the 6 catalytic subunits diluted into ELISA buffer: β5 diluted 1∶5000; β1, LMP7 and LMP2 diluted 1∶2000; β2 diluted 1∶3000; and MECL1 diluted 1∶1000. The beads were washed 5 times with 200 µl/well of ELISA buffer and incubated with HRP-conjugated secondary antibody: goat anti-rabbit for β5 diluted 1∶2000, rabbit anti-goat for MECL1 diluted 1∶5000, and goat anti-mouse for LMP7 and LMP2 diluted 1∶5000, and goat anti-mouse for β1 and β2 diluted 1∶2000 in ELISA buffer and incubated for 2 hrs at room temperature on a plate shaker. The beads were washed 5 times with 200 µl/well ELISA buffer and developed for chemiluminescence signal using the supersignal ELISA pico substrate (Pierce) following manufacturer's instructions. Luminescence was measured on a plate reader (Tecan) and converted to ng of proteasome or µg/ml of lysate by comparison with the 20S proteasome or untreated cell lysate standard curves. Curve fits were generated using a sigmoidal dose response equation (Y = Bottom + [(Top-Bottom)/(1+10∧(LogEC50-X)*HillSlope)where X is the logarithm of concentration and Y is the response. For proteasome inhibitor studies, active site probe binding values were expressed as the percent of binding relative to DMSO-treated cells.

### Sequencing of the catalytic subunits

β5, β1, LMP7, LMP2 and β7 subunits were sequenced through MCLAB (South San Francisco, CA). The open reading frame of each subunit was amplified from the cDNA and subcloned into a sequencing vector for analysis.

### In silico modeling

All modeling was performed using the Molecular Operating Environment (MOE) software with default energy minimization parameters. The Cys63Phe mutation was modeled into the crystal structure of the α5/β5/β6 yeast proteasome subunits bound to epoxomicin (PDB# 1G65) or bortezomib (PDB# 2F16) and into the apo-form structure (PDB# 1RYP). For each model, Cys63Phe and all sidechains within 4.5 Å, were energy minimized, followed by an energy minimization of the helix containing Cys63Phe and all residues within 4.5 Å. A final energy minimization was performed for the inhibitor bound models including the helix, the inhibitor molecule, and all side chains within 4.5 Å. The mutant structure files have been graphically represented in Pymol.

### aCGH array and GEP analysis

Cells were cultured continuously in bortezomib for ∼1 month and harvested for gene array analysis. One fraction was lysed in TRIzol (Invitrogen) and RNA was isolated with PureLink micro-midi columns (Invitrogen) following the manufactures' recommendations. DNA was isolated from the second fraction with the Puregene kit (Qiagen; Valencia, CA). RNA quality was assessed with an Agilent BioAnalyzer and samples with RIN >9.0 were labeled and hybridized to HG-133Plus-2.0 GeneChips (Affymetrix; Santa Clara, CA). Gene expression levels were extracted from the raw data using GCRMA package in bioconductor (www.bioconductor.org) and detection estimates were calculated using MAS5 in Expression Console (Affymetrix). DNA samples were digested with DNAseI (Ambion; Foster City, CA) and the fragmented DNA was labeled with CY5-dUTP using the BioPrime Plus Labeling kit (Invitrogen). Labeled samples were competitively hybridized to SurePrint G3 Human CGH 1 M microarrays (Agilent; Santa Rosa, CA). Copy number abnormalities were identified in Genomic Workbench V5 (Agilent) using the ADM-2 algorithm and regions of variation between wild-type and resistant populations were identified. These data are MIAME compliant and the raw data has been deposited into the MIAME complaint GEO database.

### Statistical analysis

For comparisons of treatment groups, unpaired t-test (Mann-Whitney), paired t-tests, and one-way or two-way ANOVA (where appropriate) were performed. For ANOVA, Bonferroni post hoc analysis was used to compare treatment groups. All statistical analyses were performed using GraphPad Prism Software (version 4.01). Other differences were assessed by Student *t* tests. Differences of *p*<0.05 were considered significant.

## Results

### Generation of HT-29 clonal isolates conditioned under bortezomib pressure

**Figure 1:**
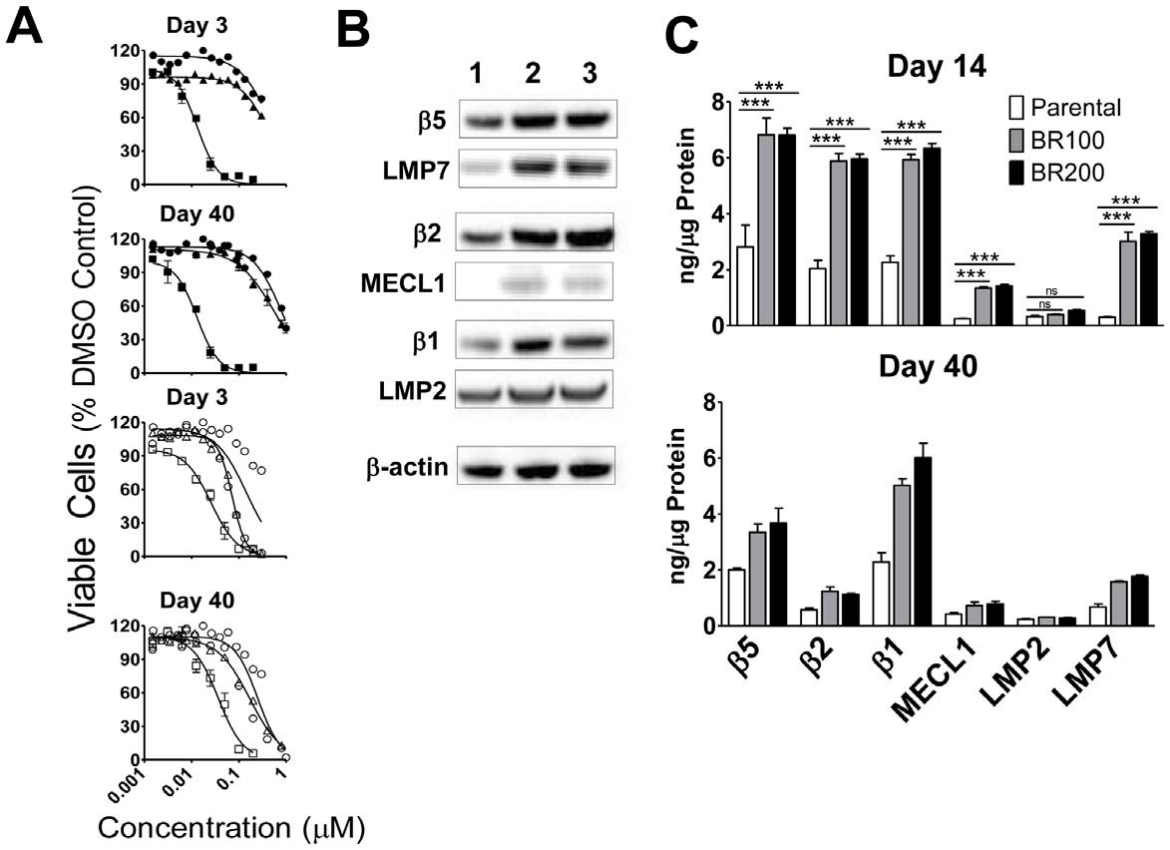
Altered proteasome expression in bortezomib-resistant HT-29 cells. (A) Parental (▪,□), BR100 (▴, Δ), and BR200 cells (•,○) were cultured for 3 or 40 days in the absence of drug prior to exposure to varying concentrations (1 nM–1 µM) of bortezomib (closed symbols) or carfilzomib (open symbols) for 72 hrs. Viability was normalized to DMSO controls and data are presented as mean viability of triplicate cultures (± S.E.M). Data is from 1 of 3 replicate experiments with similar results. (B) Western blot analysis of constitutive and immunoproteasome active site subunits in parental (1), BR100 (2), and BR200 (3) cells cultured in the absence of drug for 14 days. β-actin or GAPDH was used as an to verify equal loading. (C) Constitutive and immunoproteasome subunit levels (ng subunit/µg total protein) in parental, BR100 and BR200 cell lines cultured in the absence of drug for 14 or 40 days were measured using ProCISE. Data at day 14 are presented as mean ± S.D. of 3 independent experiments. *** = *P*<0.001 by one-way ANOVA followed by Bonferroni's Multiple Comparison Test. Data at day 40 is representative of one experiment.

To generate bortezomib-resistant cells, HT-29 cells were cultured in bulk with continuous step-wise increases in bortezomib concentration. Cells were grown in the presence of 20 nM bortezomib (1 month), followed by 60 nM (1 month), then 100 nM (1 month) and 200 nM (1 month). Clonal isolates were then derived by limiting dilution culture in the presence of 100 nM (BR100) or 200 nM (BR200) bortezomib for an additional 4 months to establish stable resistance. Three clones were isolated for each conditioning concentration (100 and 200 nM) and all of the clones were similar in their resistance to bortezomib as determined by viability studies (Figure 1A). When kept under constant pressure, the resistant clones showed similar morphologies to the parental line by light microscopy. However, the resistant cells displayed slower growth rates and were qualitatively less adherent when compared to the parental cell lines (data not shown). When cultured without bortezomib for up to 40 days, the resistant clones displayed similar adherent properties and doubling times as the parental cells.

### Resistance in BR100 and BR200 is stable and specific to bortezomib

In parental cells, bortezomib induced cell killing with a 50% inhibitory concentration (IC_50_) of 0.013 µM. Compared to parental cells, BR100 and BR200 cells were 26-63-fold more resistant to the cytotoxic potential of bortezomib following culture for 3–40 days in the absence of bortezomib (Figure 1 and Table 1). The BR100 and BR200 lines were 26-34-fold resistant to bortezomib when assessed for cell viability at 3 (1 passage) or 14 days (4 passages) after culture without bortezomib (Table 1). Following 20 days of culture in the absence of bortezomib, the level of resistance to bortezomib increased to 45-fold and by 40 days had increased to 63-fold in the BR200 cell line, while similar levels of bortezomib resistance were maintained in the BR100 cells.

**Table 1:** Cell viability of HT-29, BR100 and BR200 cell lines over a 40-day period.

	Parental		BR100				BR200			
Days removed from BTZ	IC_50_ (µM)		IC_50_ (µM)		Fold Resistance		IC_50_ (µM)		Fold Resistance	
	BTZ	CFZ	BTZ	CFZ	BTZ	CFZ	BTZ	CFZ	BTZ	CFZ
**3**	**0.013**	**0.026**	**0.41**	**0.07**	**32×**	**2.8×**	**0.44**	**0.1**	**34×**	**3.8×**
	(0.010–0.018)	(0.024–0.034)	(0.36–0.48)	(0.069–0.075)			(0.34–0.59)	(0.10–0.20)		
**14**	**0.011**	**0.031**	**0.34**	**0.09**	**26×**	**2.9×**	**0.36**	**0.11**	**28×**	**3.5×**
	(0.011–0.016)	(0.023–0.033)	(0.30–0.37)	(0.077–0.093)			(0.30–0.44)	(0.099–0.12)		
**20**	**0.015**	**0.035**	**0.42**	**0.09**	**28×**	**2.6×**	**0.68**	**0.15**	**45×**	**4.3×**
	(0.012–0.018)	(0.026–0.037)	(0.32–0.54)	(0.069–0.11)			(0.61–0.74)	(0.13–0.17)		
**40**	**0.013**	**0.037**	**0.62**	**0.17**	**48×**	**4.6×**	**0.82**	**0.15**	**63×**	**4.1×**
	(0.011–0.015)	(0.025–0.042)	(0.52–0.72)	(0.16–0.21)			(0.74–0.90)	(0.14–0.17)		

The mean IC_50_ values are presented with 95% confidence intervals from 2 independent experiments in parenthesis.

Additionally, we tested the resistant cell lines for sensitivity to other proteasome inhibitors. Carfilzomib is a tetrapeptide epoxyketone that irreversibly inhibits the proteasome CT-L subunits and has equimolar cytotoxic potential against parental HT-29 cells as does bortezomib (Figure 1 and Table 1). Over the 40 day period, carfilzomib demonstrated relatively potent cytotoxicity in both the BR100 and BR200 cells, with a resistance factor of <5 at all time points. These data suggest that inhibition in these solid tumor cells can be overcome by other classes of proteasome inhibitors.

### Bortezomib-resistant cell lines have increased proteasome levels and activity

To assess any changes in proteasome levels in the resistant cell lines, cells cultured for 14 days without bortezomib pressure were monitored for levels of c20S (β5, β2, β1) and i20S (LMP7, LMP2, and MECL1) as analyzed by western blotting and quantitatively confirmed by ProCISE, an active site probe-based proteasome subunit ELISA (Figure 1B–C). Western blot analysis of the BR100 and BR200 cells showed an increase in levels of all 3 subunits of the c20S. Changes in immunoproteasome subunit expression were more varied. LMP7, which was expressed at a low level in the parental cells, showed a dramatic increase in both resistant cell lines. Similarly, MECL1, which was undetectable in the parental cells, was increased in the both the BR100 and BR200 lines. Conversely, LMP2, which was detectable in the parental lines, did not show altered expression in the resistant cells.

To enumerate the changes in proteasome levels, we utilized ProCISE to quantitate active site subunit levels in the 3 cells lines after 14 days of culture in the absence of drug (Figure 1C). When compared to parental cells, there was a 3-4-fold increase in all 3 c20S active site subunits. LMP7, which was expressed at 10–30% of the levels of β5 in parental cells, showed an 8-fold increase in expression in both resistant cell lines. Similarly, MECL1, demonstrated a 3-4-fold increase in resistant cells. In contrast, LMP2, showed no increase in expression level in the resistant cells. Culturing resistant cells for 40 days in the absence of bortezomib resulted in normalization of β2 and MECL1 levels and a reduction in the expression of β5 and LMP7, though the levels of these subunits remained elevated as compared to parental cells. Interestingly, levels of β1 remained constant during the culture period. When the resistant cells were subsequently re-cultured under continuous bortezomib exposure, the relative levels of subunits increased in the resistant cells (data not shown). To determine if the increased levels of proteasome active site subunits altered catalytic activity, we utilized substrate based assays to measure chymotrypsin-like (LLVY-AMC), caspase-like (LLE-AMC) and trypsin-like (VGR-AMC) activities in cells cultured for 3 days in the absence of drug. All three proteolytic activities were increased by 3-4-fold in the resistant cells as compared to the parental cells (Table 2). Taken together, stable resistance to bortezomib results in increased levels of both constitutive and immunoproteasome active sites that are further increased by continuous drug exposure.

**Table 2:** Proteasome catalytic activity of HT-29, BR100 and BR200 cells.

Substrate	LLVY		VGR		LLE	
Subunit	β5 and LMP7		β2 and MECL1		β1 and LMP2	
	Activity	Fold Change	Activity	FoldChange	Activity	FoldChange
Parental	16.5±8.25	-	27.5±3.12	-	28.4±4.24	-
BR100	54.7±5.32	3.3×	97.0±6.56	3.5×	114±10.7	4.0×
BR200	57.8±6.33	3.5×	92.5±8.10	3.3×	123±5.69	4.3×

Values presented are mean specific activity (µM AMC released/µg protein) ± S.D. from 2 independent experiments.

### Genetic characterization of HT-29, BR100 and BR200 cells

To profile gene copy number and expression changes resulting from bortezomib resistance, array CGH (aCGH) and gene expression analysis (GEP) were performed on all 3 cell lines. When compared to parental HT-29 cells, we identified 59 genes with amplifications and 60 genes with deletions in BR100 and BR200 cells (Figure S1 and Tables S3, S4, S5, S6, S7). Copy number alterations were noted in genes that belong to the transcription and translation, transporters, cell signaling, differentiation and proliferation families of proteins. By expression analysis, 3134 genes displayed a 2-fold or greater change in gene expression in both BR100 and BR200 cells (Table S1), including multiple genes that have been previously reported to be linked with proteasome inhibition and/or bortezomib resistance (Table S2).

No deletions or amplifications were identified for genes encoding proteins found in the 26S proteasome. However, changes in gene expression levels, which were consistent with increased subunit levels described above were noted. β5 and β2 expression levels were higher in the resistant cells, but mRNA levels of β1 and the active sites of the immunoproteasome subunits did not show a greater than a 2-fold increase. We also detected increases in structural subunits of the proteasome, including β4,β6, α2, and α3, and regulatory subunits C1, C3IP, C5, C6, D1, D11, and D12.

In addition, we sequenced 4 active site subunit genes, β5, β1, LMP7 and LMP2, and one structural subunit (β7) from multiple clones in each of the 2 resistant cells lines and compared them to the sequence of the subunits in the parental cells. We chose these active sites due to the reported activity of bortezomib against these proteasome activities [22]. β7 was analyzed since it is the critical structural subunit required for assembly of a complete 20S proteasome particle [2]. All resistant clones had a mutation in the propeptide region (Arg24Cys) and a mutation (Cys63Phe) within the active site region of β5. In addition, 2 out of the 3 BR200 clones had a Phe50Ile mutation in the propeptide regions of LMP7. No mutations were found in β1, β7, or LMP2. Taken together, these data demonstrate that resistance to bortezomib is correlated with genomic alterations that affect gene expression levels and that specific point mutations are selected in the β5 gene.

### Bortezomib-treated and resistant cells have more rapid recovery of proteasome activity

**Figure 2:**
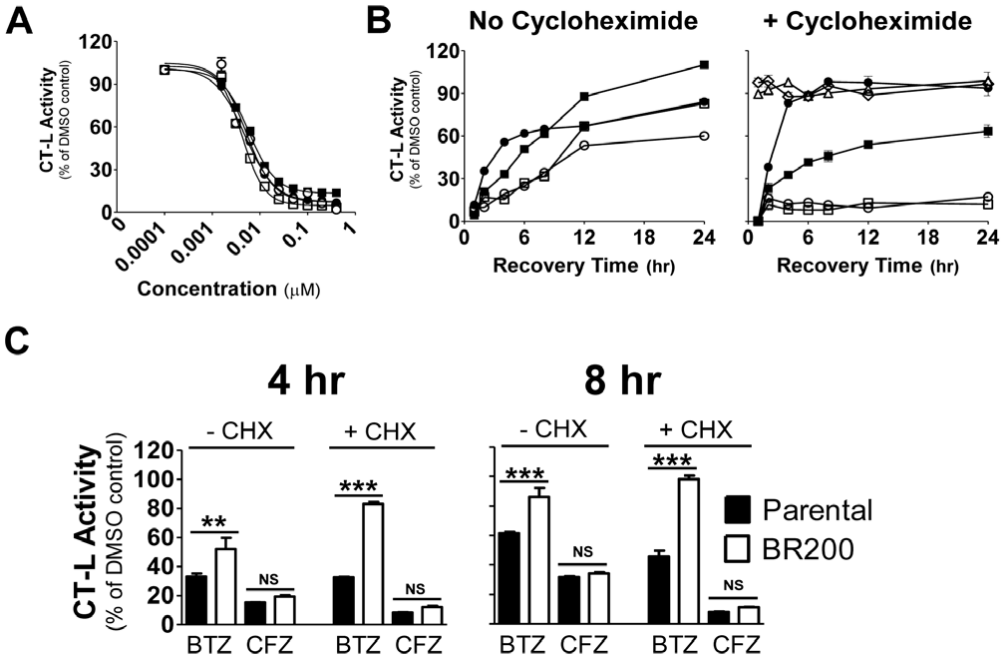
Increased proteasome turnover in bortezomib-resistant cells. (A) Parental (▪,□) and BR200 cells (•,○) were cultured for 3 days in the absence of drug prior to exposure to varying concentrations (1 nM–1 µM) of bortezomib (closed symbols) or carfilzomib (open symbols) for 1 hr. Proteasome chymotrypsin-like activity was measured using LLVY-AMC as substrate and specific activity values were normalized to DMSO controls. Data are presented as the mean relative activity (± S.E.M.) and is representative of 2 replicate experiments. (B) Parental (▪,□) and BR200 cells (•,○) were exposed to 100 nM bortezomib (closed symbols) or carfilzomib (open symbols) for 1 hr, washed and cultured in drug free media with or without cycloheximide for 1, 2, 4, 6, 8, 12 , and 24 hr prior to measurement of chymotrypsin-like activity. Parental cells (Δ) and BR200 (◊) cells treated with CHX alone in the absence of drug are included as additional controls. Data are presented as the mean relative activity (± S.E.M.) and is representative of 2 replicate experiments. (C) Relative chymotrypsin-like activity in parental and BR200 cells at 4 or 8 hr after a 1 hr pulse exposure to 100 nM bortezomib or carfilzomib in the presence or absence of cycloheximide. ** = *P*<0.01; *** = *P*<0.001 by one-way ANOVA followed by Newman-Keuls post-hoc comparisons.

In order to determine if the gene expression changes or point mutations in proteasome active sites resulted in altered proteasome activity, we measured the inhibitory activity of bortezomib and carfilzomib against the proteasome CT-L activity in parental and resistant cells. Both compounds resulted in equivalent levels of proteasome inhibition 1 hr after exposure in both the BR100 and BR200 cell line (Figure 2A; Figure S2A).

Next, we exposed the cells to 100 nM of either compound, a concentration resulting in near complete inhibition of CT-L activity, for 1 hr prior to washing and culturing in drug free media for 24 hr. A subset of cells were cultured in the presence of cycloheximide to block protein translation. CT-L activity was measured at various time points for recovery of proteasome activity during the 24 hr culture period. We noted that proteasome activity in the resistant cells exposed to bortezomib recovered more quickly in the first 8 hr as compared to the parental cells (Figure 2B and C; Figure S2B and C). This recovery was not due to new proteasome production since similar effects were seen in the presence of cycloheximide. Carfilzomib showed less recovery of proteasome inhibition as compared to bortezomib over the 24 hr culture period in parental and resistant cells. The recovery of CT-L activity following brief exposure to carfilzomib was similar in resistant and parental cells. When cells were exposed to carfilzomib and allowed to recover in the presence of cycloheximide, an initial recovery of ∼20% of total activity was noted after 1 hr of culture. However, no further recovery of proteasome activity was noted in carfilzomib treated cells.

We tested 2 other proteasome inhibitors in our resistant cell lines to further explore the mechanism of proteasome inhibition in the setting of bortezomib resistance. Leu-Leu-Leu-Aldehyde (MG132), a peptide aldehyde and completely reversible inhibitor displayed 8–13 fold resistance with 3 days out of constant bortezomib pressure (Figure S3). In contrast, no differences in the potency of proteasome inhibition, kinetics of recovery of proteasome activity, or cytotoxic potential of Leu-Leu-Leu-Boronate (LLL-Bor) were noted in a batch cell culture of HT-29 cells resistant to 100 nM bortezomib (Figure S4). In addition, we determined that this inhibitor, like carfilzomib, is an irreversible proteasome inhibitor, as determined by monitoring proteasome recovery in the presence of cycloheximide.

Since sensitivity to LLL-Bor was maintained, de-boronating enzymes are unlikely to be responsible for the observed resistance and rapid proteasome activity recovery with bortezomib. Indeed, no de-boronating enzymes such as cytochrome P450s were found to be upregulated in the gene expression data (Tables S1, S2, S3, S4, S5, S6, S7).

Finally, to eliminate the possibility of bortezomib off-target activity on serine proteases contributing to the resistance mechanism, we evaluated their expression in the resistant cell lines. Although cathepsin G, cathepsin A, DPP2 and HtrA2 (chymase was undetectable by western blotting) were found to be upregulated compared to parental cells after 3 days of culture without bortezomib pressure, by 14 days of drug free growth, protein expression levels of these serine off-targets reached similar levels as compared to parentals (Figure S5). While the immediate upregulation of the off-target serine proteases may be expected due to bortezomib exposure, the re-establishment of baseline levels over time demonstrates that these off-targets are not involved in the mechanism of resistance in the BR100 and BR200 cells that remain stably resistant to 40 days.

### Cys63Phe mutation in the β5 subunit is critical for bortezomib binding stability

**Figure 3:**
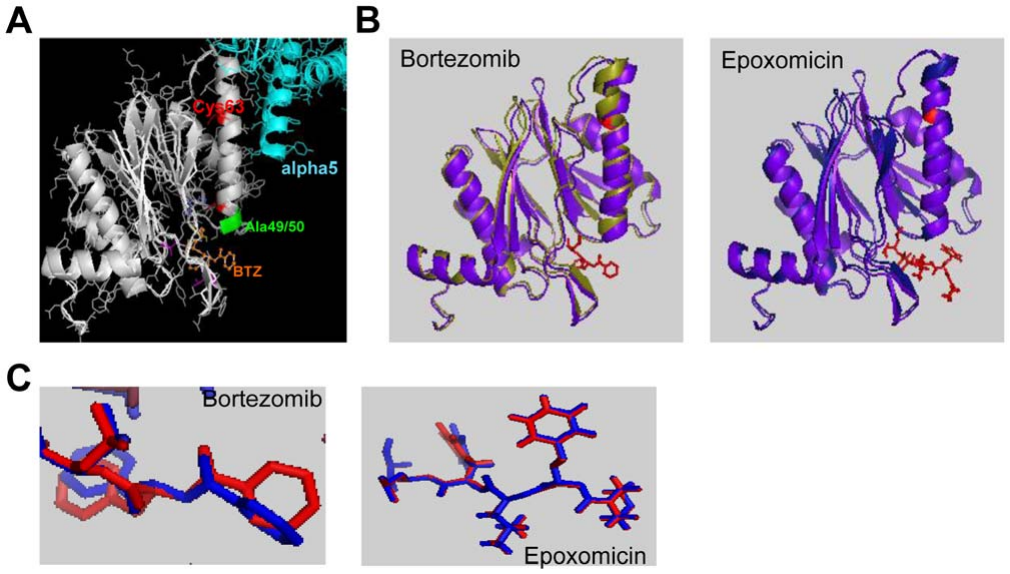
Cys63Phe mutation is a critical structural mutation. (A) Cys63Phe mutation is part of a critical helix at the α5 (cyan)/β5 (gray) subunit interface subunit that directly points into the active site, with Ala49/50 making direct contacts with proteasome inhibitors. (B) Mutant PRE unbound (purple) overlaid with mutant PRE bound to bortezomib (olive) and epoxomicin (midnight blue). (C) Active site view of bortezomib and epoxomicin bound to the wild-type (blue) and mutant (red) PRE2 structures.

The altered rates of recovery of CT-L activity in bortezomib resistant cells and the presence of a mutation in the active site region of β5 suggested a conformational change in the binding site structure of this subunit. In order to understand the impact of the Cys63Phe mutation, we modeled the mutation in the crystal structures of the yeast proteasome α5/β5/β6 subunits unbound (apo) or bound to one of 2 inhibitors, bortezomib and epoxomicin, an epoxyketone related to carfilzomib [21]. Since human β5 shares ∼84% homology with the yeast gene, we used the yeast subunit to model the mutations rather than building a homology model of the human β5 based on the yeast structure in order to conserve the integrity of the crystal structure as much as possible. Cys63 is housed in the same helix as Ala49/50, residues critical for bortezomib binding [23] (Figure 3A). Based on our model, the Cys63Phe mutation leads to a shift in the angle of the helix with respect to the active site (Data not shown). This shift is much more significant in the inhibitor bound forms than in the unbound form. This shift did not alter the orientation of epoxomicin but resulted in a twist in the orientation of bortezomib (Figure 3C). As a result, the conformation of bortezomib within the active site was shifted (Figure 3C). Furthermore, bortezomib binding in the Cys63Phe mutant resulted in a larger shift of the helix than noted with epoxomicin (Figure 3B). Taken together, our modeling results indicate that the Cys63Phe mutation affects the position of the helix and, therefore, likely affects the binding of bortezomib to β5.

## Discussion

The introduction of bortezomib to the armamentarium of myeloma therapy has resulted significant clinical success but primary refractoriness and treatment-emergent drug resistance has deprived a subset of patients of effective therapy [7], [8]. To date, in vitro models of bortezomib resistance have resulted in mutations in the primary target of bortezomib (β5) that have not yet been described in bortezomib treated patients [12]–[14]. In order to better understand the mechanism of drug resistance, we developed multiple lines of solid tumor HT-29 cells adapted to continuous bortezomib pressure. These cells displayed approximately a 30-fold resistance when compared to parental cells and resistance increased to 60-fold after long-term culture without bortezomib. By using ProCISE, a proteasome subunit ELISA, aCGH and GEP analysis, and comparing the activity of bortezomib to another class of proteasome inhibitor, we have been able to elucidate some general themes involved in proteasome resistance. These mechanisms of resistance were generally similar to what has been previously reported with bortezomib adapted hematologic-derived tumor cell lines (increase in proteasome activity, mutations in the β5 subunit and genetic alterations in stress response and cell survival pathways), demonstrating that resistance to this dipeptide boronate proteasome inhibitor is independent of tumor cell lineage.

In this study we report novel specific alterations of the proteasome subunits in resistant cells, previously undescribed mutations in β5, including one known to occur in MM patients, and how bortezomib resistance can be overcome with irreversible proteasome inhibitors. In hematologic-derived tumor cell lines conditioned to be bortezomib resistant, western blot analysis demonstrated increased expression of constitutive proteasome subunits [11], [15], [17], [18]. In both the BR100 and BR200, we noted a 3–4 fold increase in the levels of all c20S and i20S subunits, except for LMP2. It is noteworthy that bortezomib has been shown in separate studies to potently target LMP2, including in patient derived cells [10], [24]. With a longer duration of culture following withdrawal of bortezomib, subunit expression, with the exception of β1, decreased in the resistant cells but remained elevated relative to parental cells. Since resistance to bortezomib increased during this period, these data suggest that increased expression of proteasome subunits provides only a partial explanation for drug resistance. Increased subunit levels by ProCISE correlated with increased mRNA levels as determined by microarray analysis and a 3-4-fold increase in basal activities of the three catalytic sites as has been observed before [11], [15], [17], [18]. The consistent elevation of β1 levels after 40 days, may reflect the 10-fold prolonged half-life of this protein relative to other active site subunits of the constitutive proteasome [25]. An increase in β1 levels may also explain why LMP2 levels did not increase in concordance with LMP7 and MECL1, as β1 is capable of forming hybrid proteasomes with immunoproteasome subunits [26]. It is noteworthy that lower levels of LMP2 expression correlated with resistance to proteasome inhibition in B-cell tumor lines [27].

Since bortezomib resistance increased during a period in which proteasome subunit levels decreased, we sought to determine if altered kinetics of proteasome turnover was also involved in drug resistance. Indeed, after a 1 hr pulse of bortezomib, proteasome activity recovered more rapidly in the resistant cells, in particular during the first 8 hr post drug exposure. This was not a result of more rapid production of new proteasomes since we also noted more rapid recovery of proteasome activity following pulse exposure of bortezomib in the presence of cycloheximide.

Previous studies with hematologic-derived bortezomib resistant cell lines have shown mutations at 2 key residues in the bortezomib binding site, Ala49 and 50 [11]–[13], [17]. These adjacent residues are critical for coordinating a water molecule which is required for bortezomib stabilization. However, to date, these mutations have not been identified in the β5 gene in bortezomib refractory patients [20]. In both the BR100 and BR200 clones we detected a mutation in β5 (Cys63Phe) that resides in the same alpha helix as Ala49/50, though not directly in the active site. Additionally, our structural model suggests a conformational and unfavorable shift of bortezomib within the active site. Although the contribution of Cys63Phe mutation on bortezomib binding through movement of the helix would be a long range effect, such allosteric long range effects have been previously reported between the different active site β subunits, indicating that the proteasome has a highly dynamic structure [28]. The fact that proteasome activity recovers faster in the BTZ resistance cell lines in the presence of cycloheximide suggest that Cys63Phe increases the dissociation constant of BTZ, presumably by altering the position of the alpha helix.

A second mutation found in the resistant cells (Arg24Cys) is contained within the propeptide portion of the β5 subunit. Like all proteasome active site subunits, β5 is translated as a proezyme containing a 49-amino acid propeptide portion that is cleaved prior to assembly into the β-ring of the proteasome. Previous work has shown that the portion containing Arg24 is required for proper subunit processing [2]. Expression of β5 without the propeptide is lethal in yeast but growth can be restored by expressing the propeptide as a separate transcript [29], [29,29,30]. Therefore, it is possible that the altered recovery of proteasome activity following bortezomib exposure is a result of altered β5 processing. It is noteworthy that the prevalance of Arg24Cys is 5 times higher in patients with MM than in the general population [31]. Although, expression of a β5 construct containing Cys at position 24 did not alter proteasome activity or sensitivity to the proteasome inhibitor MG262, kinetics of proteasome recovery were not assessed in that study. Of further note was the presence of a mutation in the propeptide portion of LMP7 (Phe50Ile) in 2 of 3 BR200 clonal isolates. Our data suggest that sequencing efforts of proteasome active sites in bortezomib refractory patient tumor cells may shed light on the mechanism of clinical resistance.

By both aCGH and GEP analysis, the genetic profiles of BR100 and BR200 were found to be similar. In corroboration with protein analysis, we found upregulation of several proteasome genes (catalytic, structural, and regulatory subunits) in resistant cells. However, deletion or reduced expression of several gene families was surprising since their elevated levels have been linked to bortezomib resistance. We found that co-factors of HSP70 were deleted and together with amplification of a chromosomal region containing a negative regulator of HSP70 suggests that HSP70 activity is being suppressed in resistant cells. Given that HSP70 activation is associated with bortezomib resistance in lymphoma cells [32], our results suggest that alterations in this pathway are not causal mediators of resistance. Increased expression of another heat shock protein, HSP27, was noted in BR100 and BR200 cells. Increased expression of HSP27 has been determined to be associated with BTZ in lymphoma cells in vitro and in patients refractory to bortezomib [32], [33]. Since increase levels of HSP27 were also associated with poor prognosis in dexamethasone-treated patients, this may represent a general mechanism of drug resistance.

Importantly, we determined that resistance to bortezomib could be overcome by two other proteasome inhibitors. Carfilzomib, which has demonstrated activity in bortezomib relapsed and refractory MM patients [34], showed only a 3–4 fold decrease in cytotoxic potential in the BR100 and BR200 cells. This modest change in cytotoxicity was equivalent to the increase in proteasome enzymatic activity noted in the same cells. Since carfilzomib remained sensitive in both MM and DLBCL lines made ∼5 fold resistant to bortezomib, we hypothesize that carfilzomib can overcome bortezomib resistance across multiple histotypes and varying degrees of resistance [35], [36]. Another irreversible proteasome inhibitor, LLL-Bor, was also equivalently cytotoxic to both resistant and parental cells, while the cells remained refractory to a reversible inhibitor MG132. Further, bortezomib off-target activity does not likely play a role in the resistance mechanism since there was no stable increase in expression of these proteases. The results with these compounds suggest that bortezomib resistance, and the subsequent genetic and protein expression changes, do not result in resistance to all classes of proteasome inhibitors. These data also suggest that expression of markers of multidrug resistance, such as HSP27, does not predict for resistance to carfilzomib treatment.

Carfilzomib differs from bortezomib in the mechanism of proteasome inhibition mediated by the pharmacophore. Similar to epoxomicin, carfilzomib contains an epoxyketone moiety which forms a dual covalent morpholino adduct with the N-terminal threonine of the proteasome active sites [37]. Consistent with this, we noted that there was no recovery of proteasome activity in tumor cells following a pulse treatment of carfilzomib in the presence of cycloheximide. This irreversible mechanism of inhibition results in a longer duration of proteasome inhibition as compared to bortezomib. Furthermore, in bortezomib-resistant cells, recovery of proteasome activity following a pulse treatment with carfilzomib was equivalent to that seen in parental cells. Similar findings were also seen with LLL-Bor, which we also determined to be an irreversible inhibitor. This suggests that inhibition of β5 by irreversible inhibitors such as carfilzomib is unaffected by the mutational status. Indeed, structural modeling suggests that epoxomicin binding to the β5 active site was unaffected by the mutation at position 63. Previously, we had shown that in a panel of tumor cells, carfilzomib had greater cytotoxic potential than bortezomib when both agents were exposed to tumor cells for 1 hr prior to a 72 hr culture [21]. Together with the data we report here, this suggests that the prolonged duration of proteasome inhibition achieved with carfilzomib results in a greater anti-tumor response. In conclusion, these data present the description of cell lines and assays to show the biochemical and genetic study of bortezomib resistance in vitro and that resistance to one class of proteasome inhibitor can be overcome by the introduction of potent, irreversible inhibitors.

## Supporting Information

Figure S1 **Whole Genome Comparison of HT-29 Variants.** Regions of DNA content gain and loss are shown for chromosomes 1–22, X and Y.
(TIF)

Figure S2 **Increased proteasome turnover in bortezomib resistant cells.** (A) Parental (▪,□) and BR100 cells (•,○) were cultured for 3 days in the absence of drug prior to exposure to varying concentrations (1 nM–1 µM) bortezomib (closed symbols) or carfilzomib (open symbols) for 1 hr. Proteasome chymotrypsin-like activity was measured using LLVY-AMC as substrate and specific activity values were normalized to DMSO controls. Data are presented as the mean relative activity (± S.E.M.) and is from 1 of 2 replicate experiments. (B) Parental (▪,□) and BR100 cells (•,○) were exposed to 100 nM bortezomib (closed symbols) or carfilzomib (open symbols) for 1 hr, washed and cultured in drug free media with or without cycloheximide for 1, 2, 4, 6, 8, 12 , and 24 hr prior to measurement of chymotrypsin like activity. As additional controls, parental (open triangle) cells treated with CHX alone in the absence of drug are compared to cells treated with DMSO. BR100 (◊) cells treated with CHX alone in the absence of drug are compared to parental cells treated with DMSO. Data are presented as the mean relative activity (± S.E.M.) and is from 1 of 2 replicate experiments. (C) Relative chymotrypsin-like activity in parental and BR100 cells 4 or 8 hr after a 1 hr pulse exposure to 100 nM bortezomib or carfilzomib in the presence or absence of cycloheximide. ** = P<0.01; *** = P<0.001 by one-way ANOVA followed by Newman-Keuls post-hoc comparisons.
(TIF)

Figure S3 **Effect of MG132 in parental and HT-29 resistant cells.** (A) Parental cells were cultured for 3 days with bortezomib exposure, allowed to recover for 3 days, then treated for 72 hrs with a dose range of MG132, bortezomib and carfilzomib and cell viability was assessed using CellTiter glo. Open triangles denote effect of MG132, black circles denote effect of carfilzomib and black squares represent bortezomib. (B) BR100 cells were cultured for 3 days and treated with either MG132, carfilzomib or bortezomib as described in (A). (C) BR200 cells were cultured and treated with drug as described in (A). (D) IC_50_ values for the curves in (A–C) is shown above. Data are presented as the mean relative activity (± S.E.M.) and is from 1 of 2 replicate experiments.
(TIF)

Figure S4 **Characterization of LLL-boronate in BR100 batch cells.** (A) Parental cells were cultured for 3–40 days with bortezomib exposure, allowed to recover for 3 days, then treated for 72 hrs with a dose range of bortezomib and LLL-boronate and cell viability was assessed using CellTiter glo. Square shapes denote bortezomib data and triangles denote response with LLL-boronate. The same compounds were used in a batch population of cells resistant to 100 nM bortezomib (right panel). (B) Percent chymotrypsin-like activity at the 4 hr time point for the 100 nM bortezomib dose and LLL-boronate in parental cells (left panel) and in batch cells resistant to 100 nM bortezomib (right panel). (C) Parental (▪,□) and BR100 batch cells (•,○) were exposed to 100 nM bortezomib (closed symbols) or LLL-boronate (open symbols) for 1 hr, washed and cultured in drug free media with or without cycloheximide for 1, 2, 4, 6, 8, 12 , and 24 hr prior to measurement of chymotrypsin like activity. Data are presented as the mean relative activity (± S.E.M.) and is from 1 of 2 replicate experiments.
(TIF)

Figure S5 **Serine Protease off-target activity in bortezomib-resistant cells.** BR100 and BR200 cells were cultured without bortezomib for 3 or 14 days, along with parental cells, and cells were harvested for immunoblot analysis. Either cell lysates from PBMCs or SH-SY5Y cells were used as appropriate controls. Data are representative of 2 separate experiments.
(TIF)

Table S1 **Genes with Two Fold or Greater Change in Gene Expression in both BR100 and BR200.**
(XLSX)

Table S2 **Fold Change of Genes Expressed in Wild-type and/or Resistant Cell Lines Related to Proteasome Function or Drug Resistance.**
(XLSX)

Table S3 **Genes Deleted in BR100 with a Two Fold or Greater Gene Expression Change.**
(XLSX)

Table S4 **Genes Deleted in BR200 with a Two Fold or Greater Gene Expression Change.**
(XLSX)

Table S5 **Genes Amplified in BR100 with a Two Fold or Greater Gene Expression Change.**
(XLSX)

Table S6 **Genes Amplified in BR200 with a Two Fold or Greater Gene Expression Change.**
(XLSX)

Table S7 **Summary of Tables S3, S4, S5, S6.**
(XLSX)
