# Supplementary figures and images for: Molecular Mechanisms of Bortezomib Resistant Adenocarcinoma Cells
Source: PLoS One. 2011 Dec 22;6(12):e27996. doi: 10.1371/journal.pone.0027996 (PMC3245226; doi:10.1371/journal.pone.0027996)

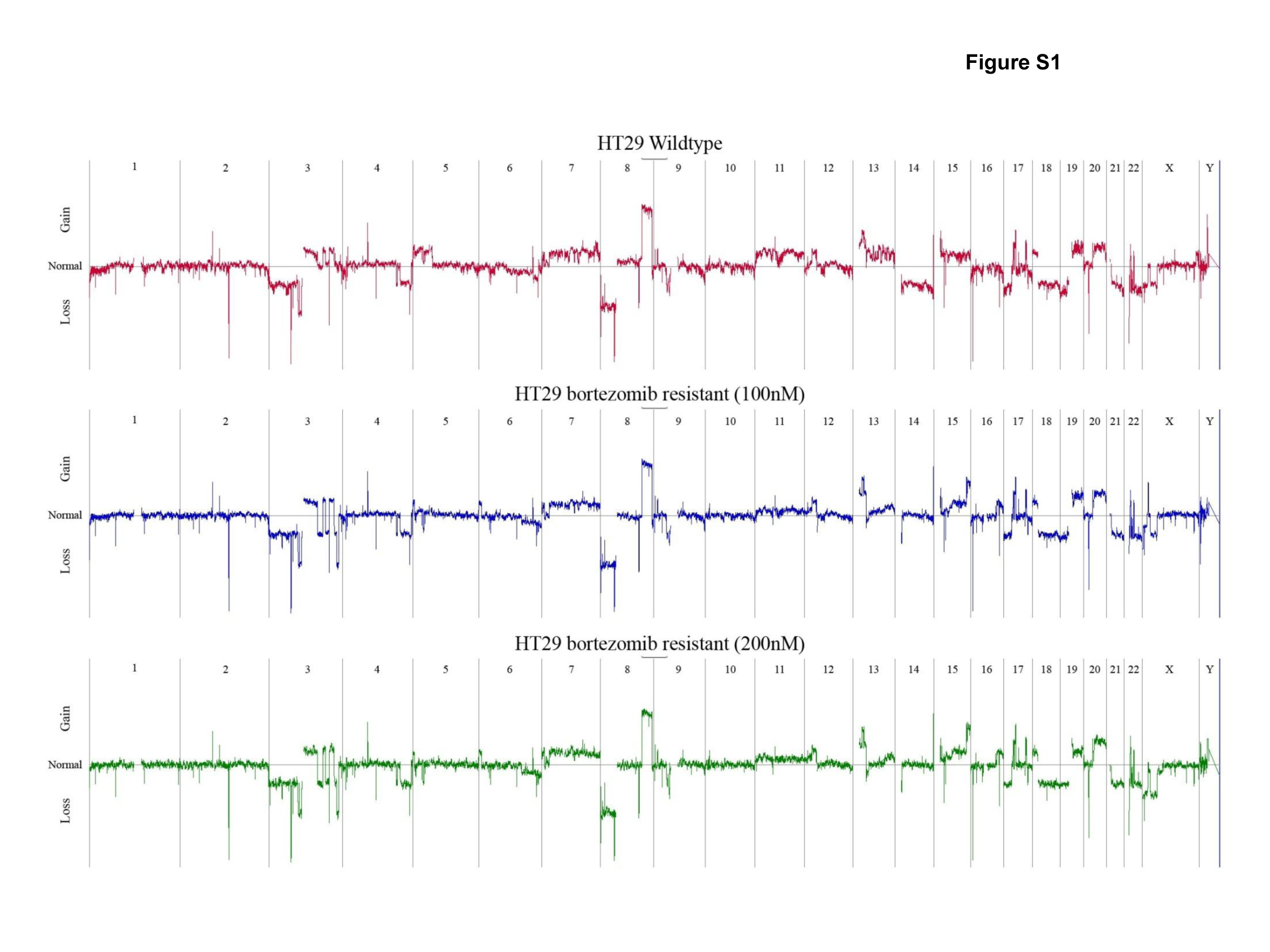

Supplement: Figure S1 — Whole Genome Comparison of HT-29 Variants. Regions of DNA content gain and loss are shown for chromosomes 1–22, X and Y. (TIF) [file pone.0027996.s001.tif]

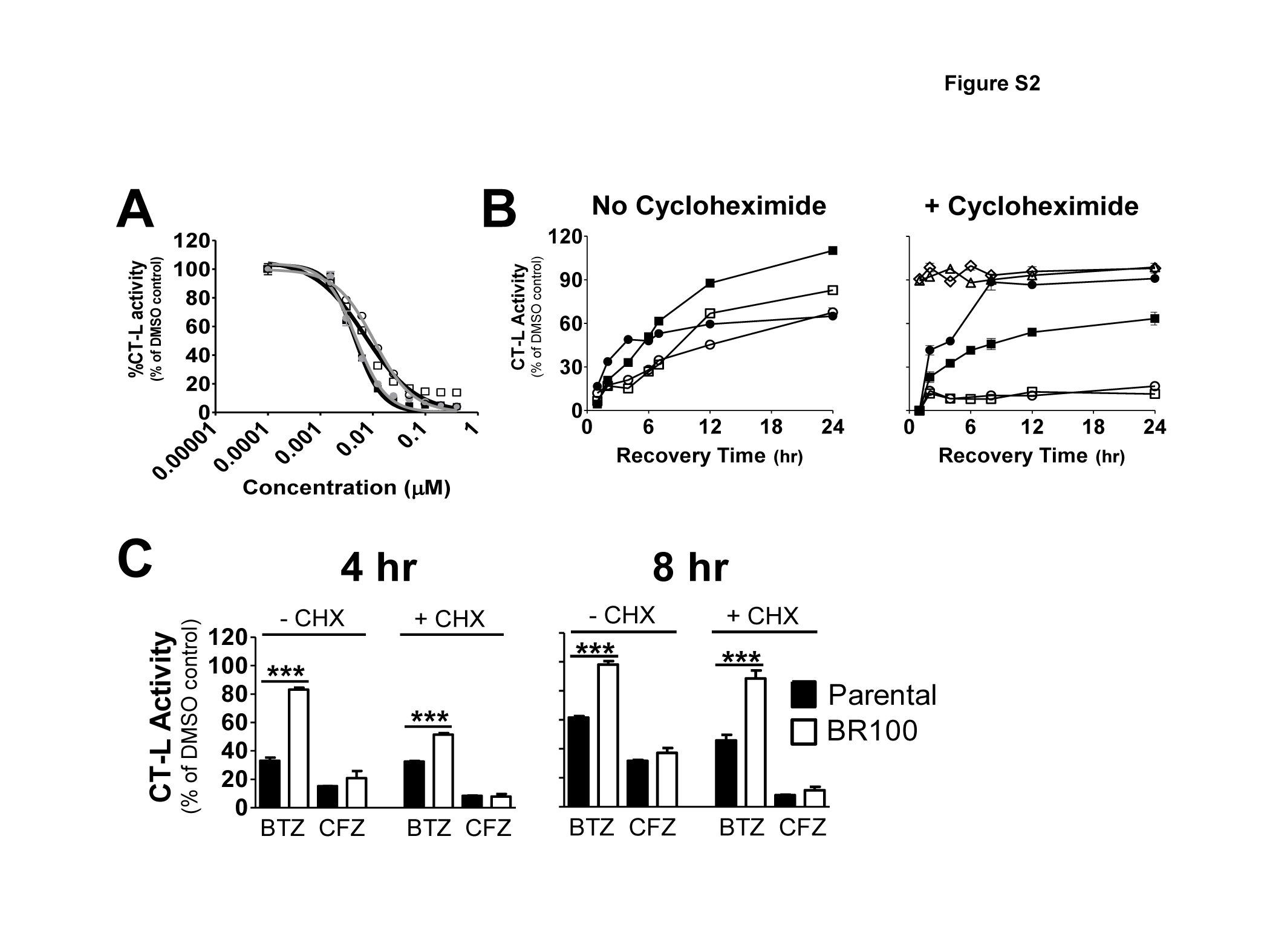

Supplement: Figure S2 — Increased proteasome turnover in bortezomib resistant cells. (A) Parental (▪,□) and BR100 cells (•,○) were cultured for 3 days in the absence of drug prior to exposure to varying concentrations (1 nM–1 µM) bortezomib (closed symbols) or carfilzomib (open symbols) for 1 hr. Proteasome chymotrypsin-like activity was measured using LLVY-AMC as substrate and specific activity values were normalized to DMSO controls. Data are presented as the mean relative activity (± S.E.M.) and is from 1 of 2 replicate experiments. (B) Parental (▪,□) and BR100 cells (•,○) were exposed to 100 nM bortezomib (closed symbols) or carfilzomib (open symbols) for 1 hr, washed and cultured in drug free media with or without cycloheximide for 1, 2, 4, 6, 8, 12 , and 24 hr prior to measurement of chymotrypsin like activity. As additional controls, parental (open triangle) cells treated with CHX alone in the absence of drug are compared to cells treated with DMSO. BR100 (◊) cells treated with CHX alone in the absence of drug are compared to parental cells treated with DMSO. Data are presented as the mean relative activity (± S.E.M.) and is from 1 of 2 replicate experiments. (C) Relative chymotrypsin-like activity in parental and BR100 cells 4 or 8 hr after a 1 hr pulse exposure to 100 nM bortezomib or carfilzomib in the presence or absence of cycloheximide. ** = P<0.01; *** = P<0.001 by one-way ANOVA followed by Newman-Keuls post-hoc comparisons. (TIF) [file pone.0027996.s002.tif]

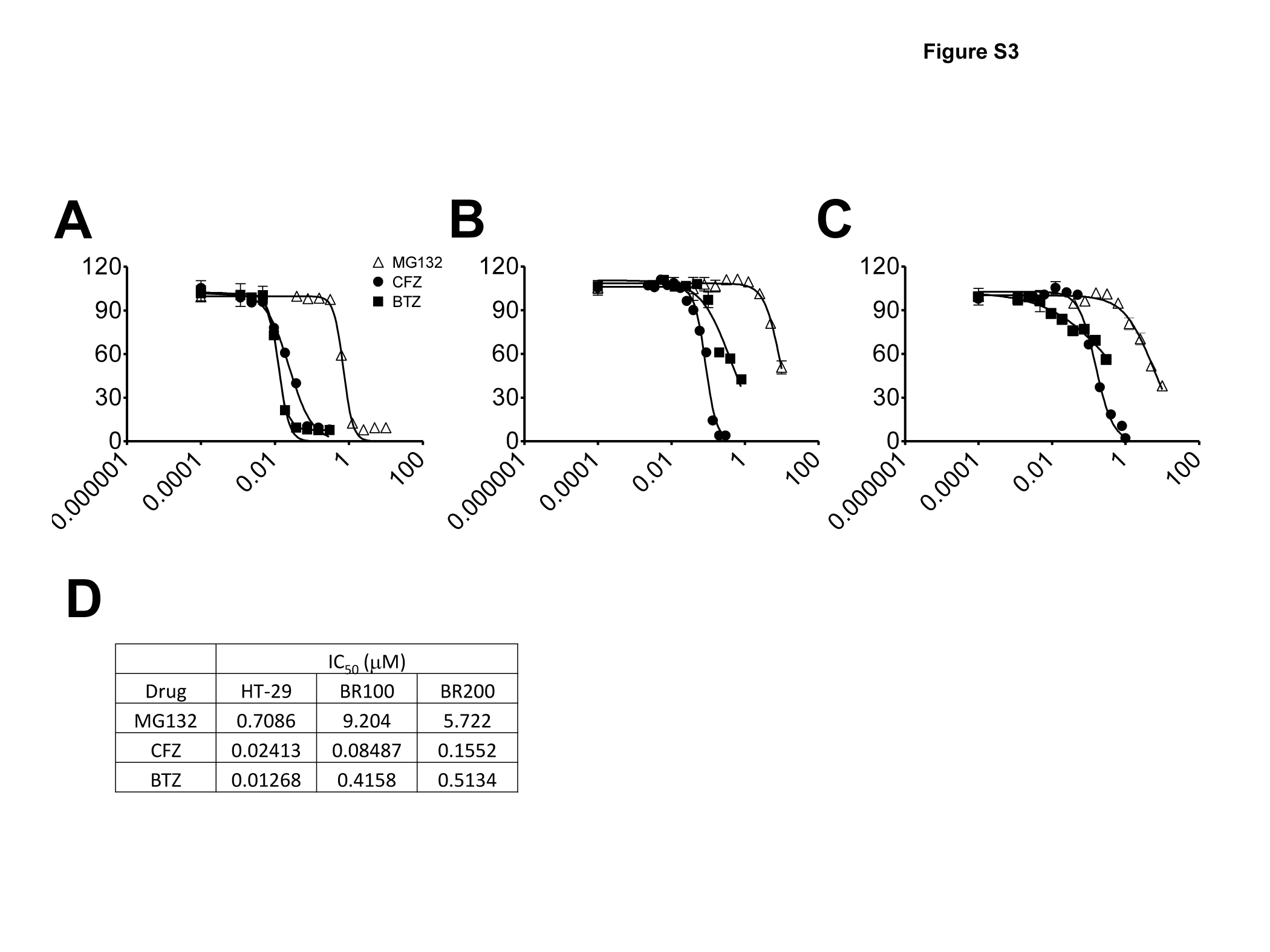

Supplement: Figure S3 — Effect of MG132 in parental and HT-29 resistant cells. (A) Parental cells were cultured for 3 days with bortezomib exposure, allowed to recover for 3 days, then treated for 72 hrs with a dose range of MG132, bortezomib and carfilzomib and cell viability was assessed using CellTiter glo. Open triangles denote effect of MG132, black circles denote effect of carfilzomib and black squares represent bortezomib. (B) BR100 cells were cultured for 3 days and treated with either MG132, carfilzomib or bortezomib as described in (A). (C) BR200 cells were cultured and treated with drug as described in (A). (D) IC50 values for the curves in (A–C) is shown above. Data are presented as the mean relative activity (± S.E.M.) and is from 1 of 2 replicate experiments. (TIF) [file pone.0027996.s003.tif]

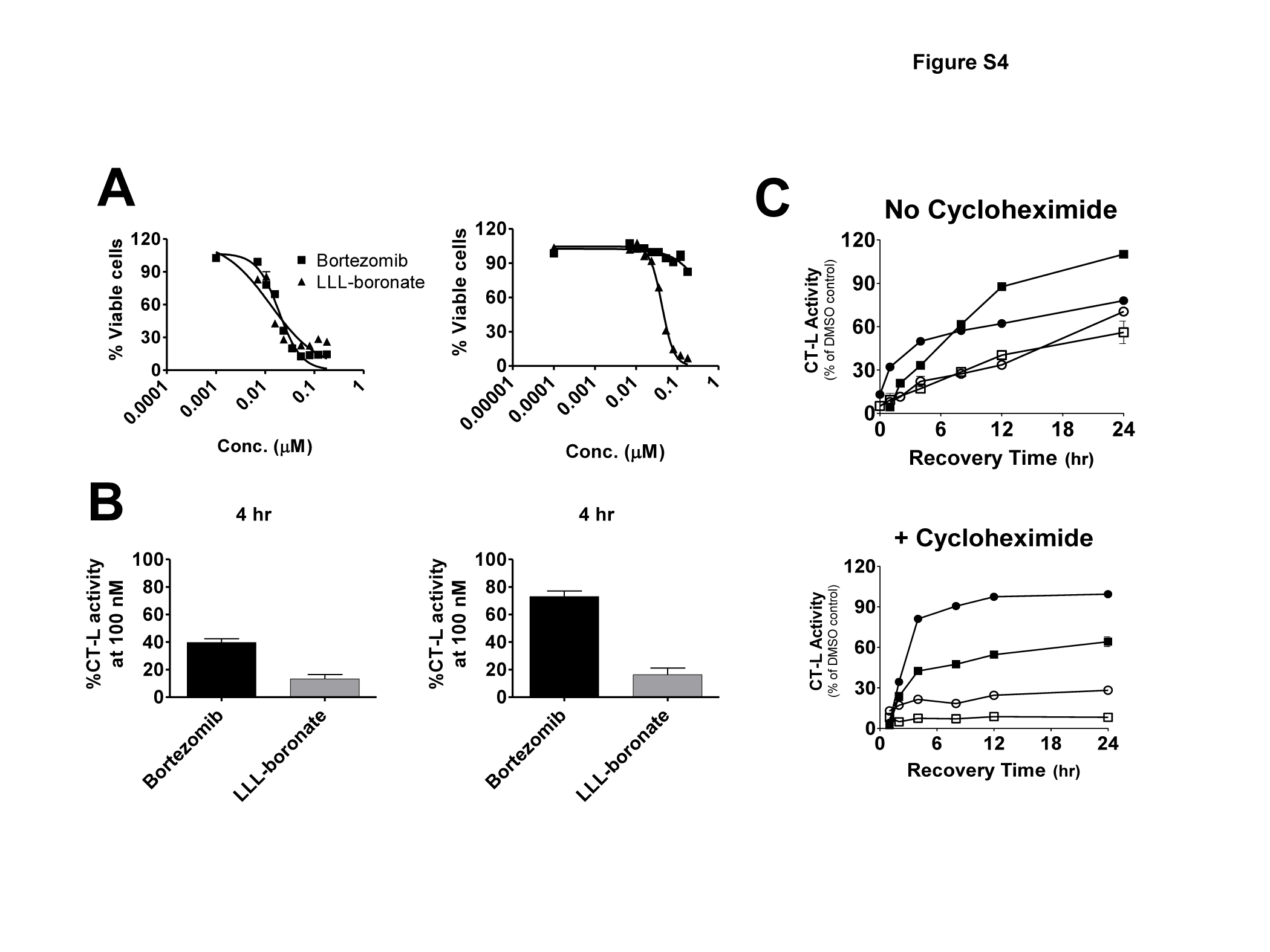

Supplement: Figure S4 — Characterization of LLL-boronate in BR100 batch cells. (A) Parental cells were cultured for 3–40 days with bortezomib exposure, allowed to recover for 3 days, then treated for 72 hrs with a dose range of bortezomib and LLL-boronate and cell viability was assessed using CellTiter glo. Square shapes denote bortezomib data and triangles denote response with LLL-boronate. The same compounds were used in a batch population of cells resistant to 100 nM bortezomib (right panel). (B) Percent chymotrypsin-like activity at the 4 hr time point for the 100 nM bortezomib dose and LLL-boronate in parental cells (left panel) and in batch cells resistant to 100 nM bortezomib (right panel). (C) Parental (▪,□) and BR100 batch cells (•,○) were exposed to 100 nM bortezomib (closed symbols) or LLL-boronate (open symbols) for 1 hr, washed and cultured in drug free media with or without cycloheximide for 1, 2, 4, 6, 8, 12 , and 24 hr prior to measurement of chymotrypsin like activity. Data are presented as the mean relative activity (± S.E.M.) and is from 1 of 2 replicate experiments. (TIF) [file pone.0027996.s004.tif]

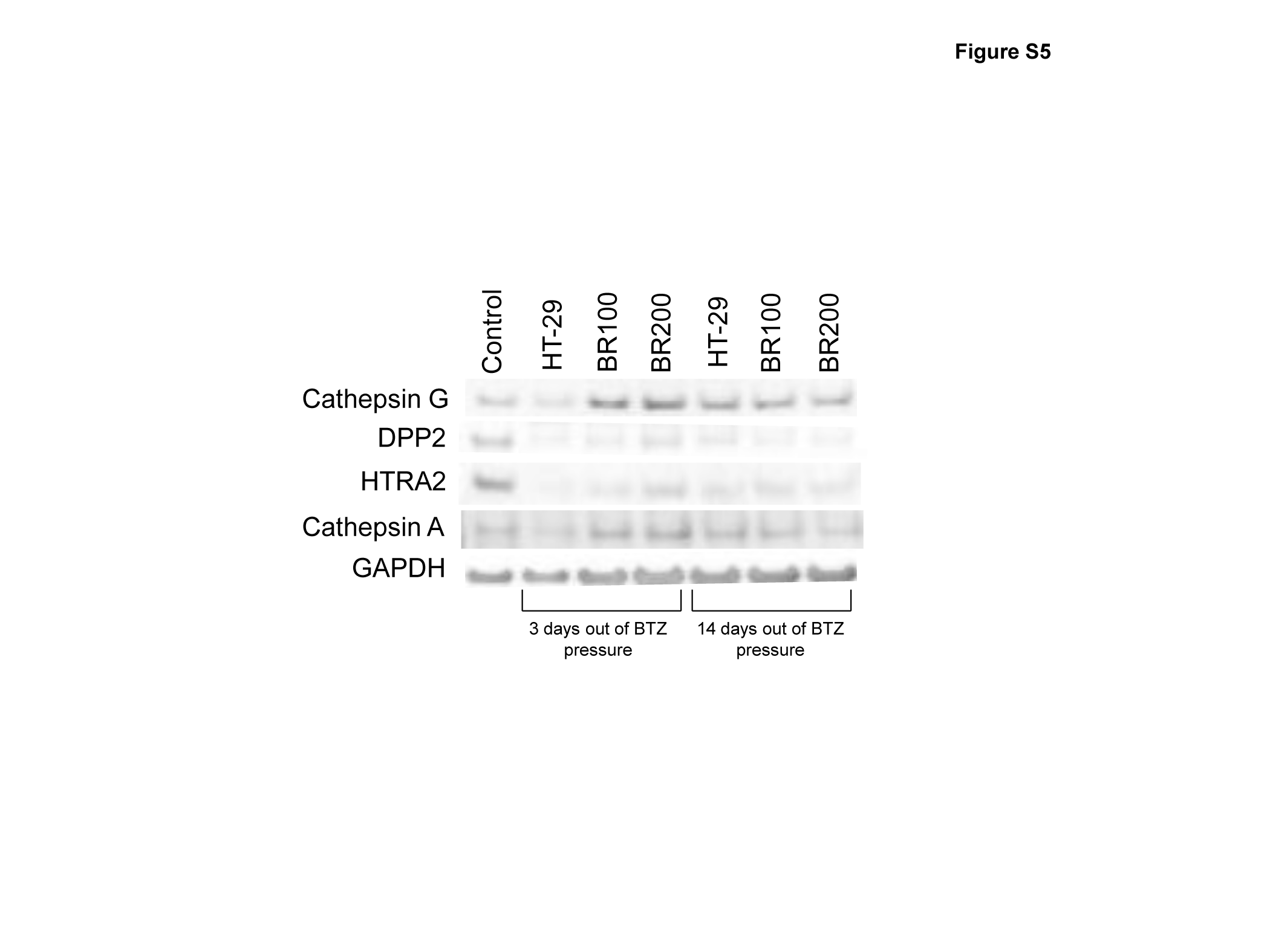

Supplement: Figure S5 — Serine Protease off-target activity in bortezomib-resistant cells. BR100 and BR200 cells were cultured without bortezomib for 3 or 14 days, along with parental cells, and cells were harvested for immunoblot analysis. Either cell lysates from PBMCs or SH-SY5Y cells were used as appropriate controls. Data are representative of 2 separate experiments. (TIF) [file pone.0027996.s005.tif]
